# Association of prescribed oral stimulants on cocaine use among patients enrolled in opioid agonist treatment: A retrospective longitudinal cohort study

**DOI:** 10.3389/fpsyt.2022.1074691

**Published:** 2022-12-02

**Authors:** Mark Tatangelo, Farah Tahsin, Kristen A. Morin, David C. Marsh

**Affiliations:** ^1^Northern Ontario School of Medicine, Greater Sudbury, ON, Canada; ^2^Institute for Clinical Evaluative Sciences (ICES) North, Greater Sudbury, ON, Canada; ^3^Dalla Lana School of Public Health, University of Toronto, Toronto, ON, Canada; ^4^Health Sciences North, Greater Sudbury, ON, Canada

**Keywords:** retrospective longitudinal study, opioid agonist treatment (OAT), observational study, oral stimulation, cocaine use disorder

## Abstract

**Objectives:**

The objective of this study was to measure the association of prescribed oral stimulants with the consumption of cocaine among a population of patients receiving Opioid Agonist Therapy (OAT).

**Methods:**

The study was a retrospective clinical cohort study using the medical records of all patients receiving OAT who attended treatment clinics within the Canadian Addiction Treatment Centers (CATC) in Ontario from April 2014 to February 2021. Linear mixed-effects models were fit for the exposure of prescribed oral stimulants, and the outcome of a positive urinalysis drug screen for cocaine. Covariates for age, sex, and a random effect for patients were fitted to account for differences between and within patient observations over time.

**Results:**

Among patients receiving OAT therapy *n* = 314 patients were prescribed oral stimulants and *n* = 11,879 patients were not prescribed oral stimulants among Ontario CATC clinics (*n* = 92, *n* = 145 physicians), the mean age at enrollment for patients receiving oral stimulants was 37.0, *SD* = 8.8, with 43.6% female patients and for patients not receiving oral stimulants mean age was 36.6, *SD* = 10.7, with 39.6% female patients. Linear mixed effects models showed no difference in cocaine-positive urine tests over time for fixed effects *B* = 0.001, however, when considering the Interclass correlation coefficient (ICC) between the fixed effects, we found that time since the prescription of an oral stimulant was associated with a decrease of ICC = −0.14 in cocaine positive urine tests. Increasing age at prescription ICC = −0.92, and being male ICC = −0.23 were associated with decreasing cocaine-positive urine.

**Conclusion:**

The use of oral stimulant prescriptions to treat cocaine use had no clinically significant benefit in a real-world setting. Patients who receive prescriptions for oral stimulants consume more cocaine before and after treatment compared to patients without an oral stimulant prescription. We also observed that cocaine use was reduced with increased time since treatment initiation.

## Introduction

Opioid use disorder continues to be a significant challenge in Ontario and worldwide (1, 2). According to the Public Health Agency of Canada report, the crude rate of total apparent opioid toxicity death in Ontario has increased over the years (3). There were 6.2 (per 100,000 population) opioid toxicity deaths in 2016 whereas the number increased to 16.4 (per 100,000 population) in 2020 (4). A large number of opioid-related toxicity death occur due to polysubstance use by individuals such as the use of cocaine and fentanyl (3).

Opioid Agonist Therapy (OAT) is currently the gold standard for patients with opioid use disorder (5, 6). In OAT, opioid withdrawal is managed by taking medications such as methadone or buprenorphine/naloxone. Although clinical guidelines recommend the use of OAT, data among specific sub-groups of patients receiving OAT is often absent or difficult to measure despite clinical interest because of differential health insurance coverage, and challenges in longitudinal follow-up of this patient population. These subgroups are of clinical interest because OAT could have variable effectiveness profiles in sub-populations, for example, those receiving oral stimulant medications and/or consuming cocaine (4, 7).

Cocaine is a stimulant that inhibits dopamine reuptake in the brain and long-term cocaine use is associated with declined cognitive functioning (8). In 2019, cocaine was the most commonly used illegal drug among Canadians, which accounted for approximately half of illegal drug use (9). Patients using cocaine while in OAT are especially concerning because 30–50% of OAT enrollees self-report cocaine use (10, 11). A previous study conducted by our research group showed that individuals in OAT who use cocaine have a lower retention rate in the treatment and early treatment discontinuation (4, 7). Additionally, a previous study in the United States has identified that OAT patients who regularly use cocaine are at increased risk of overdose (12). Therefore, interventions that reduce the consumption of cocaine among OAT patients could also improve treatment efficacy, outcomes, and persistence on OAT.

There is no pharmacological treatment available for cocaine use disorder (13). Recently, the use of oral stimulants for cocaine use disorder has gained some traction due to a small number of clinical trials conducted (14, 15). Among oral stimulants that have been considered promising to treat cocaine use disorder, bupropion and dextroamphetamine are considered to be effective for achieving sustained cocaine abstinence, according to a Cochrane Collaboration review of psychostimulant drugs (16). Because of the interaction between substance use, prescription of oral stimulants, and OAT, it is difficult to separate the association of these factors when they may exert complex or interlocking effects. Therefore, the use of a longitudinal clinical cohort with repeated measurements over time of both urinalysis testing and medication dispensation (OAT and oral stimulants) is needed to support limited trial evidence with observational data from a real-world clinical setting. Since the data collected was from patients with identical health insurance coverage, the confounder of multiple insurers was removed which could explain these complex relationships to investigate solutions that can help with reducing the frequency of cocaine use among OAT patients and improve patient persistence. The objective of this study was to measure the association of prescription oral stimulant medications on cocaine consumption among a population of patients receiving opioid agonist treatment.

## Materials and methods

### Study design and setting

The study was a longitudinal clinical cohort study with repeated measurements using the medical records of all patients receiving oral stimulants and already enrolled in OAT at The Canadian Addiction Treatment Centers (CATC) in Ontario from April 2014 to February 2021. In Ontario, the inclusion Manual version V (17). Ontario has a single-payer healthcare system, whereby all residents have identical health care coverage under the Ontario Health Insurance Plan (OHIP) with access to OAT. The CATC is the largest network of addiction medicine clinics in Canada (approximately 70 clinics across Ontario). CATC provides comprehensive care for patients who have substance use disorder which includes pharmacological therapy, primary care, harm reduction, and counseling. Standardized practices, policies, and operating procedures within the clinic network, limit the likelihood of treatment variability between sites.

### Participants and data sources

Enrollment criteria for the CATC were a Opioid Use Disorder requiring treatment with an OAT (including methadone and buprenorphine/naloxone), and patient age > = 18 years of age. No minimum follow-up date was enforced for this study, but patients must have at least one valid urinalysis test.

Participating CATC clinics were in Ontario with physicians and patients consenting to share data for research use. Data were collected using the EZMethPro electronic medical record system (18) with data available for each visit from the first visit until the end of follow-up or up to a maximum follow-up date of February 28, 2021. Urinalysis testing for opioids and controlled substances were conducted for each patient at ratio randomization intervals generated automatically by the electronic medical record system across the patient population. Patients with a first prescription of amphetamine/dextroamphetamine, methylphenidate, lisdexamfetamine, modafinil, dextroamphetamine sulfate, or cetirizine were identified as cases, with an index date of first prescription and a 365-day washout period without an oral stimulant observable in the cohort. The oral stimulants were prescribed using standard Health Canada dosing guidelines (18). Patients without an eligible prescription of oral stimulants were eligible controls during their follow-up time at the same age and sex as the case patients.

### Variables

The study outcome was a positive urinalysis test for cocaine metabolite, defined as a urinalysis threshold value greater than 100 ng/ml (19). Study exposure was the first prescription of oral stimulant.

Covariates included in the analysis were age, sex, and cocaine use history prior to the index date of first oral stimulant prescription with all covariates collected from the electronic medical record. Additional, clinical characteristics were urine drug screening (UDS) results for fentanyl, cannabis, and opioids. Data were collected from identical sources for both exposed and unexposed patients.

### Statistical methods

**FIGURE 1:**
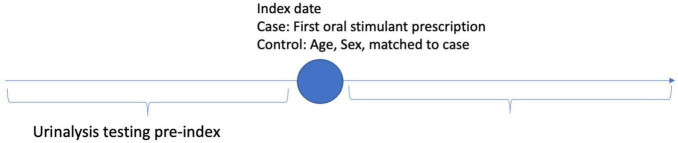
Model 1-Pre-post prescription among cases and controls index date.

**FIGURE 2:**
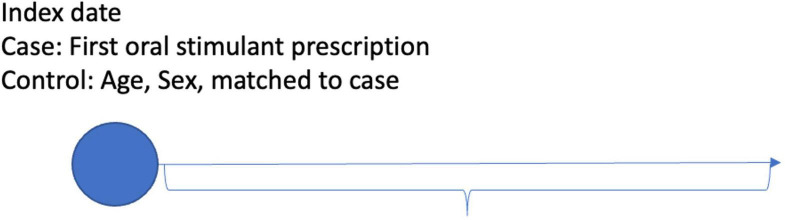
Model 2-Follow up only.

**FIGURE 3:**
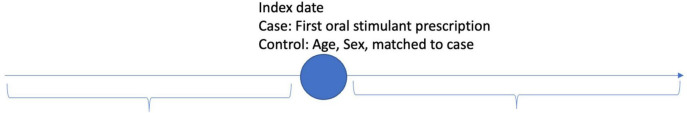
Model 3-Pre-post prescription among cases only.

The data were analyzed using descriptive statistics of mean and standard deviation for all demographic, time-dependent confounding and non-independence of observations within and between patients. Random effects were fit for each patient to adjust for the within-patient differences over time (20). Differential follow-up time was implicitly controlled using random effects. Subgroups and interaction effects between covariates were examined using ANOVA for omnibus differences and interclass correlation coefficient matrices (ICC) for pairwise differences in covariates. Time before and after the index event of prescription of oral stimulants was operationalized by calculating the number of days before or after the index event for patients receiving an oral stimulant, and for controls without oral stimulant patients were compared who had been matched on age, sex, and year of cohort entry to cases (Figure 1). Three models were fit to analyze the longitudinal data. Model 1 compared patients with an oral stimulant prescription to those without a before and after the index date to compare the association of patients with an oral stimulant prescription to those without before and after a prescription. Model 2 compared patients with an oral stimulant to those without an oral stimulant using post-index date follow-up only (Figure 2). Model 3 compared within patient differences among those who received an oral stimulant before and after prescription (Figure 3). All fixed effects estimates used a threshold for statistical significance of p < 0.05.

Time-to-event models using Kaplan-Meier were fit to assess the time to the first cocaine-positive urine test from the index date of patients receiving an oral stimulant.

Missing data for medications were not present in the dataset because missingness would occur because of lack of collection, not a recorded missing observation. Patient observations with invalid or inconclusive urine tests were excluded and not considered missing because these are clinically valid results. No other missing data was present because the database structure requires entry to be complete.

Study ethics were obtained from the research ethics board at the Laurentian University Research Ethics Board. Analysis of data and results were produced using R statistical software version 4.1.1 and the packages lme4 for linear mixed effects models (21). The Strobe Reporting Guidelines 2021 version for observational cohort studies was applied to this study.

## Results

Among patients receiving OAT therapy, *n* = 1,067 patients were prescribed oral stimulants at any time and *n* = 29,210 patients were not prescribed oral stimulants among Ontario CATC clinics (*n* = 92, *n* = 145 physicians). The mean age at enrollment for patients receiving oral stimulants was 37.0, *SD* = 8.8, with 43.6% female patients and for patients not receiving oral stimulants mean age was 36.6, *SD* = 10.7, with 39.6% female patients (Table 1). Patients who started in the cohort without an oral stimulant but then received an oral stimulant (*n* = 314) had 13,043 observed urine tests. Matched non-oral stimulant prescribed controls on age, sex, and year of cohort entry (*n* = 11,867) had 550,526 observed urine tests (Table 1). Linear mixed effects regression comparing patients with and without prescription of oral stimulants (Model 1) showed no statistical difference in cocaine-positive urine tests before and after prescription (*B* = 0.00467, *SD* = 0.00588, *P* = 0.427) (Table 2). However, when considering the ICC between the fixed effects, we found that time since the prescription of an oral stimulant was associated with a decrease of ICC = −0.09 in cocaine-positive urine tests (Table 3). Examining follow-up data only (Model 2), not adjusting for pre-prescription differences we found a statistically significant increase in cocaine-positive urine tests (*B* = 0.0873, *SD* = 0.0162, *P* < 0.01). Among the patients who received oral stimulants (Model 3), the pre-post analysis showed a non-statistically significant decrease in cocaine-positive urine tests (*B* = −0.00829, *SD* = 0.00787, *P* = 0.292). Within-patient variance values of 0.765 for models 1 and 2 indicate approximately 7% of the variance in outcomes are observed within patients over time. However, the variance within patients increases to 0.0904 among the group receiving oral stimulants only showing greater variance in positive urine tests within patients over time.

**TABLE 1:** Patient demographics.

Variable mean (SD)	Exposure	
Exposure group	Oral stimulant group (*n* = 314)	Control (*n* = 11,879)
Patient years of follow-up	173.2	7628.9
Number of urinalysis tests	13,043	550,546
Number of positive tests (%)	4,068 (31.2)	109,492 (19.9)
Urinalysis test per patient	41.54	46.35
Positive tests per patient	12.95	9.21
Age	37.0 (8.8)	36.6 (10.7)
Number female (% female)	137 (43.6)	4,706 (39.6)

**TABLE 2:** Model results.

	Beta coefficient	Std.	*P-value*

Model 1			
**Fixed effects**			
(Intercept)	2.54E-01	9.50E-03	<0.01
Time (days)	−5.34E-05	4.17E-06	<0.01
Oral stimulant	4.67E-03	5.88E-03	0.427
Gender (M)	5.70E-03	5.34E-03	0.285
Age (years)	−2.06E-03	2.44E-04	<0.01
Random effects		Variance	Std. dev.
Within patient	(Intercept)	0.07658	0.2767
Residual		7.55E-02	0.2747

**Model 2**			

(Intercept)	2.54E-01	9.60E-03	<0.01
Time (days)	−.03E-05	4.58E-06	<0.01
Oral stimulant	8.73E-02	1.62E-02	<0.01
Gender (M)	7.41E-03	5.41E-03	0.17
Age (years)	−2.11E-03	2.46E-04	<0.01
Random effects		Variance	Std. dev.
Within patient	(Intercept)	0.07637	0.2763
Residual		7.44E-02	2.73E-01

**Model 3**			

(Intercept)	2.87E-01	5.88E-02	1.42E-06
Time (days)	−1.17E-05	1.54E-05	0.447
Oral stimulant	−8.29E-03	7.87E-03	0.292
Gender (M)	−1.31E-02	2.68E-02	0.625
Age (years)	−.79E-04	1.52E-03	0.654
Random effects		Variance	Std. dev.
Within patient	(Intercept)	0.09045	0.3008
Residual		0.11023	0.332

**TABLE 3:** Between covariate interclass correlation coefficient matrix.

	(Intr)	Time (days)	Oral stimulant	Gender (M)
Time (days)	–0.033			
Oral stimulant	–0.009	–0.097		
Gender (M)	–0.229	0.004	0.002	
Age (years)	–0.901	–0.006	–0.001	–0.116
Time (days)	–0.048			
Oral stimulant	–0.044	0.005		
Gender (M)	–0.228	0.003	0.015	
Age (years)	–0.898	–0.011	–0.008	–0.118
Time (days)	0.068			
Oral stimulant	–0.078	–0.407		
Gender (M)	–0.205	0.028	–0.007	
Age (years)	–0.928	0.063	–0.025	–0.059

Results from the other model predictors indicate that age, and gender, are not associated with cocaine-positive urine tests in any of the models. Time in days after the prescription was found to be statistically significantly associated with cocaine-positive urine tests in models 1 (*B* = −0.0000534, *SD* = 4.17E-6, *p* < 0.01) and model 2 (*B* = −0.0000603, *SD* = 4.58E-6, *p* < 0.01) showing that over time, patients are less likely to test positive for cocaine.

The results of the ICC analysis show that oral stimulants alone (ICCModel 1 = −0.009) and time (ICCModel 1 = −0.033) are both associated with decreasing cocaine-positive urine. However, non-female gender over time was associated with increases in cocaine-positive urine tests (ICC Model 1(male, time) = 0.004). Among patients who received prescriptions for oral stimulants, the male gender was correlated with higher numbers of positive tests over time ICC (male, time) = 0.028.

**FIGURE 4:**
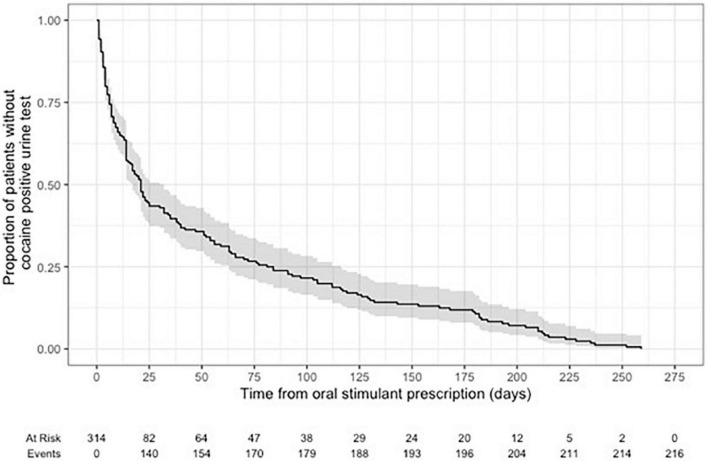
Time to first positive cocaine urine test from the first prescription of oral stimulant.

Kaplan-Meier curves for time to first positive cocaine urine test from the first prescription of oral stimulant showed 29.4% of patients tested positive at 7-days post-prescription, 42.7% positive at 14-days post prescription, and 56.5% positive after 28-days post prescription (Figure 4).

## Discussion

This study measured the association of prescription oral stimulant medications on cocaine consumption among a population of patients receiving OAT. As well as to explore the association of prescribed oral stimulants with the retention of OAT. Drawing on longitudinal data from CATC, the largest network of addiction medicine clinics in Canada, using three different statistical models, we found that prescribing oral stimulants to OAT patients was either associated with a small increase or no statistically significant effect in reducing cocaine use. Our results from observational data were unable to confirm clinical trial findings which have shown that prescribing oral stimulants to a small cohort of patients in a controlled setting was associated with reduced cocaine use (15, 22). We sought to test these hypotheses in a real-world setting with a large cohort of patients (*n* = 12,193) with many observed urine tests (563,589), and patient-years of follow-up (7,802.1). Therefore, our findings may help clinicians with decision-making regarding prescribing oral stimulants to treat cocaine use.

In all statistical models used in this study, we identified correlations with increasing age and time since prescription in reductions in cocaine-positive urine tests. The findings from this study support our previous research and existing literature indicating that concurrent drug use indicates poor outcomes, but that the effect is reduced with increased time in treatment (4, 7, 23).

Our ICC model also indicated that, among patients who received prescriptions for oral stimulants, the male gender was correlated with higher numbers of positive tests over time. This observation also aligns with the literature indicating that the prevalence of stimulant use is higher among males (22–24). This observation is particularly important in the era of increased exposure to synthetic opioids such as fentanyl, treatment retention has been declining (25–27). OAT has become more accessible to high-risk patients, including those who continue to use cocaine while in treatment (1, 10, 28). Therefore, the decreasing retention may not be reflective of the effectiveness of OAT, but a reflection of the changing needs of this population. Understanding the changing needs of the OAT population may help clinicians and policymakers in planning and recommending more patient-centered interventions.

The first statistical model used in this study compares patients with a prescription of oral stimulant to those without, before, and after the prescription index date. Our analysis shows that patients with an oral stimulant prescription test positive for cocaine more often per unit of time, compared to those without an oral stimulant in the pre-prescription time period but these results did not meet the threshold of statistical significance. We then wanted to examine if removing controls for past cocaine use increased the differences between the stimulant prescribed and control groups. Therefore, ignoring the pre-existing cocaine-positive test differences between the two groups in the second model, we showed that the difference between oral stimulants prescribed patients and non-oral stimulant patients was statistically significantly different in follow-up only. Our third model restricted the analysis to patients receiving an oral stimulant using a pre-post prescription analysis, to show the association of prescription on pre and post-prescription cocaine-positive urine tests. In this case, we found no statistically significant difference meaning the oral stimulant prescription had no association on change in cocaine-positive urine before compared to after prescription.

Our results suggest the oral stimulant intervention has no detectable effect on cocaine use in a real-world setting. It is possible that this subgroup of OAT patients experiences more exposure to physiological and social events that trigger increased drug use, and that there may not be a quick solution to reducing the use of cocaine. Large scale meta-analysis and systematic reviews have shown that patient outcomes improve when the complete package of treatment are included in the treatment of substance use disorders such as contingency management approaches to take-home doses of OAT, psychosocial supports, services to address concurrent mental and physical health, trauma-informed and culturally appropriate services (13, 29). Therefore, more research is needed to explore effective treatment options for higher-risk patients enrolling in OAT, such as those included in this study.

Some limitations in the current study require attention. First, there is a possibility of data entry and reporting errors associated with using secondary data. Second, the data is collected for purposes other than research, therefore we were limited to using the data which is routinely collected. Third, although we considered various factors in our statistical models, there is potential for unmeasured confounding, including confounding related to social and interpersonal factors, lack of measurement of addiction severity and some clinical characteristics. Lastly, because of the challenge in diagnosing ADHD in the presence of active cocaine use, and absence of standardized testing for ADHD in a searchable format within the EMR, we have included all patients prescribed oral stimulants while on OAT. We are not able to stratify asked on presence or absence of ADHD symptoms but all the prescriptions have been done with the goal of decreasing cocaine use.

## Conclusion

The use of oral stimulant prescription among patients receiving OAT showed no statistically significant difference in cocaine consumption in a real-world setting, despite modest positive effect sizes demonstrated in previously conducted clinical trials. This finding highlights the value of further investigation and understanding of the needs of patients who use cocaine while in OAT. We also observed that cocaine use was reduced with increased time since treatment initiation. Given the high rates of cocaine use among patients in OAT, our findings are important to help clinicians make informed decisions about appropriate treatments and to increase OAT retention for this group of patients. Our findings suggest a need to develop a more comprehensive strategy to treat people with concurrent substance use and opioid use disorders to maximize the benefits of OAT.

## Data availability statement

The data analyzed in this study is subject to the following licenses/restrictions: The datasets used during the current study are not publicly available due to privacy reasons, but aggregated data are included in this published article. Requests to access these datasets should be directed to MT, tatangelomark@gmail.com.

## Ethics statement

The studies involving human participants were reviewed and approved by the Laurentian University Research Ethics Board. Written informed consent for participation was not required for this study in accordance with the national legislation and the institutional requirements.

## Author contributions

MT participated in the data acquisition, conceptualization, data analysis, and final revision of the manuscript. FT participated in the conceptualization, writing of the original draft, revisions, and final revision of the manuscript. KM participated in the conceptualization, study design, supervision, and final revision of the manuscript. DM played a leadership role in the planning of the study, contributed to the interpretation of results, and final review of the manuscript. FT and KM contributed to the writing of the manuscript. All authors read and approved the final manuscript.
